# FKBP51: A Promising Therapeutic Target for Tauopathies

**DOI:** 10.1002/alz.093126

**Published:** 2025-01-03

**Authors:** Niat T Gebru, Jennifer Guergues, Stanley M. Stevens, Danielle Gulick, Laura J. Blair

**Affiliations:** ^1^ University of South Florida, Tampa, FL USA; ^2^ Byrd Alzheimer’s Center and Research Institute, Tampa, FL USA; ^3^ James A. Haley Veterans Hospital, Tampa, FL USA

## Abstract

**Background:**

Tau accumulation, a hallmark of Alzheimer’s disease, fuels cognitive decline and neuronal death. Our team identified FKBP51, a stabilizer of neurotoxic tau oligomers. Notably, FKBP51 levels increase with age and further in AD brains, where it is found associated with pathological tau. FKBP51 has also been linked to vulnerability in excitatory neurons in the AD brain and poorer cognitive stability. In mice, the absence of FKBP51 has been shown to be protective from stress‐induced deficits. Recognizing this critical role, we are now developing methods to lower FKBP51 and understand the detailed molecular effects of its removal.

**Method:**

We designed and synthesized locked‐nucleic acid (LNA) GapmeR ASOs targeting conserved regions in *FKBP5*, the gene that encodes FKBP51. The ASOs were tested in cultured cells and the brains of wild‐type mice. We also performed proteomic profiling of aged FKBP51 KO mice compared to young and aged wild‐type controls using a trapped ion mobility spectrometry (TIMS)‐QTOF instrument (timsTOF Pro, Bruker).

**Result:**

LNA GapmeR ASOs significantly lowered FKBP51 levels using less than10 nM doses with no signs of toxicity. *In vivo*, greater than 25% reductions of FKBP51 were found in the initial studies. Proteomic profiling revealed preservation of specific proteins and pathways in a young‐like state in mice lacking FKBP51.

**Conclusion:**

ASOs targeting FKBP51 show promise as a potential therapeutic avenue for reducing tau. The silencing of FKBP51 long‐term may directly reduce tau phosphorylation and aggregation, and indirectly protect neurons from tau‐mediated damage. Further optimization *in vivo* will commence shortly, followed by assessments in tau transgenic mice.

